# SARS-CoV-2 vaccination in androgen sensitive phenotypes – A study on associated factors for SARS-CoV-2 vaccination and its adverse effects among androgenetic alopecia and benign prostate hyperplasia patients

**DOI:** 10.3389/fimmu.2022.919958

**Published:** 2022-09-02

**Authors:** Zhihua Fan, Shixin Duan, Fangfen Liu, Wei Shi, Ziye Yang, Ruiyang Bai, Tao Li, Jingxian Chen, Hongfu Xie, Ji Li, Yan Tang

**Affiliations:** ^1^ Department of Dermatology, Xiangya Hospital, Central South University, Changsha, China; ^2^ Hunan Key Laboratory of Aging Biology, Xiangya Hospital, Central South University, Changsha, China; ^3^ National Clinical Research Center for Geriatric Disorders, Xiangya Hospital, Central South University, Changsha, China; ^4^ Xiangya School of Medicine, Central South University, Changsha, China

**Keywords:** SARS-CoV-2 vaccine, androgen sensitivity, AGA, BPH, public health, vaccine hesitancy, antiandrogen therapy

## Abstract

**Background:**

Androgen sensitivity, which was established as the leading etiology of androgenetic alopecia (AGA) and benign prostatic hyperplasia (BPH), plays an important role in SARS-CoV-2 infection. Vaccination is essential for AGA and BPH patients in view of the high risk from SARS-CoV-2 infection.

**Purpose:**

We aimed to investigate the associated factors for SARS-CoV-2 vaccination and its side effects in populations with AGA and BPH.

**Method:**

We collected the data on SARS-CoV-2 vaccination and adverse reactions of male AGA and BPH patients visited the outpatient of Xiangya hospital by telephone and web-based questionnaires. Vaccination rate and adverse reactions were compared by different vaccine types and use of anti-androgen therapy.

**Result:**

A total of 457 AGA patients and 397 BPH patients were recruited in this study. Among which, 92.8% AGA patients and 61.0% BPH patients had at least the first dose of SARS-CoV-2 vaccination (p < 0.001). Having comorbidities and use of anti-androgen therapy increased the risk of un-vaccination among AGA by 2.875 and 3.729 times, respectively (p < 0.001). Around 31.1% AGA patients and 9.5% BPH patients presented adverse reactions, which were mostly mild. Anti-androgen therapy increased the inclination of injection site pain after vaccination (18.7% vs 11.9%; OR: 1.708, 95% CI: 1.088-2.683, p = 0.019).

**Conclusion:**

Co-existence of other systemic diseases and anti-androgen therapy were the limiting factors for SARS-CoV-2 unvaccination, especially in AGA patients. The importance of SARS-CoV-2 vaccines should be strengthened and popularized in androgen sensitive phenotypes.

## Introduction

The COVID-19 pandemic is an ongoing global pandemic of severe acute respiratory syndrome coronavirus 2 (SARS-CoV-2), which has brought a sustained impact on public health (1–3). A high percentage of the patients with SARS-CoV-2 infection might develop life-threatening pneumonia, acute respiratory distress syndrome, or even multi-organ failure (4). More than 25 million infections and 5.1 million cumulative deaths caused by the COVID-19 pandemic were reported up to November 2021 (https://covid19.who.int/table), resulting in immense healthcare stress and tremendous public health cost (5). Hence, researches on developing an effective SARS-CoV-2 vaccine and its widespread application are crucial to terminating this pandemic.

SARS-CoV-2 vaccines were divided into various types according to different preparation technology (6). Three types of them were approved and used in China, consisting inactivated vaccines, adenovirus vector vaccines, and recombinant protein vaccines (7). However, far more efforts should be made to improve the current status of vaccination that merely 56% of the world population has received at least one dose of a SARS-CoV-2 vaccine, while only 7.1% of people in low-income countries have received at least one dose of vaccine according to the data published on December 13th on ourworldindata.org. Recently, increasing researches focused on exploring the influence factors for SARS-CoV-2 vaccine hesitancy, defined as delayed receipt or refusal of vaccines despite the availability of vaccination services (8), which affected the establishment of herd immunity remarkably. The health status of participants was found negatively correlated with vaccination willingness, that people in poor health are more resistant to vaccination. It may be explained by a growing awareness of vaccine side effects and the increasing public doubts about the safety and reliability of SARS-CoV-2 vaccines (9).

Androgen signaling has been proposed to mediate the pathophysiology of SARS-CoV-2 infection and considered as a promising treatment target recently. Angiotensin-converting enzyme 2 (ACE2), which is highly expressed in vital involved organs including lungs, the heart, and kidneys, has been proved crucial in binding and internalization of the viral spike protein during SARS-CoV-2 infection (10). 5 alpha-reductase is required to produce active metabolites of testosterone. Hence, 5 alpha-reductase inhibitors, which modulate ACE2 levels, have been promising in the treatment of SARS-CoV-2 infection, indicating the key status of androgen signaling as well (11, 12). In addition, androgen receptors have been found working as transcription promoters for transmembrane protease serine 2 (TMPRSS2), which was utilized as the host entry for SARS-COV2 virus spike proteins, suggesting an essential role of androgen sensitivity plays in SARS-COV2 infection (13). Androgen related mechanism accords with the phenomenon that men (approximately 60.3%) are more inclined to be infected with SARS-CoV-2 and they presented a higher mortality rate (14). As reported, COVID-19 mortality was 1.27 ± 0.17 times higher in male than in female according to a previous study recruited approximately 32 million COVID-19 cases in the United States (15). Sharing a similar androgen related pathway, androgenetic alopecia (AGA) and benign prostatic hyperplasia (BPH) are considered as classic phenotypes of androgen sensitivity. They have been found closely correlated with SARS-CoV-2 infection, and they presented more severe COVID-19 symptoms and higher rates of hospitalization due to COVID-19 infection (13, 16). The Gabrin sign was defined as a phenomenon that severe AGA was associated with worse hospital outcomes (17). Simultaneously, SARS-CoV-2 infection may worsen BPH symptoms and increase the incidence of BPH complications (18). Therefore, prevention from SARS-CoV-2 infection, by either vaccines or anti-androgen therapies, is urgently needed for AGA and BPH patients based on the high infection risk and poor prognosis.

Numerous studies and guidelines have been published on the management of SARS-CoV-2 vaccines among patients with psoriasis, systemic lupus erythematosus, some other autoimmune systemic diseases, and those under immune-suppressive therapies since the vaccine is considered to be a possible influence factor to aggravate primary diseases through intervening immune system (19–21). However, SARS-CoV-2 vaccination in androgen-sensitive phenotypes has been despised.

In order to clarify the current SARS-CoV-2 vaccination situation of AGA and BPH patients and its possible associated factors, we conducted a retrospective study through telephone and web-based questionnaires to better educate and promote vaccination in androgen-sensitive phenotypes.

## Method

### Study design and participants

**Figure 1:**
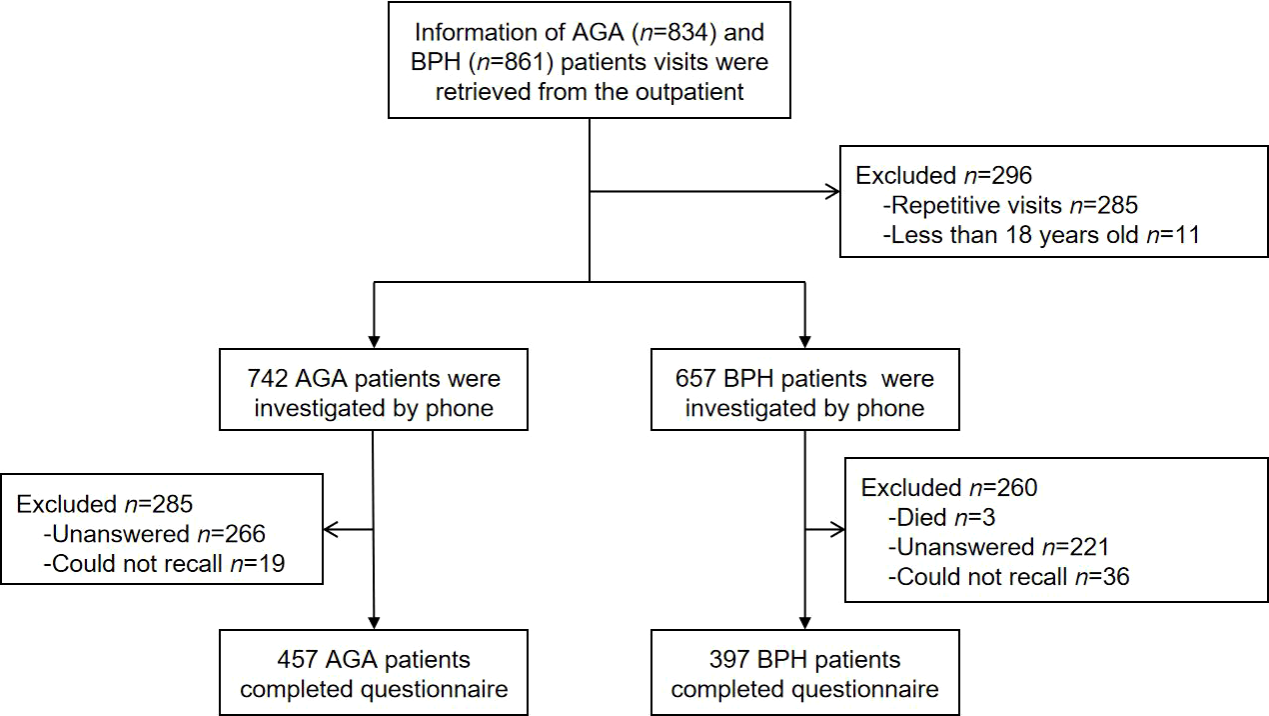
Flow diagram showing the AGA and BPH patients enrolled in the study. AGA, androgenetic alopecia; BPH, benign prostatic hyperplasia.

This is a retrospective, single-center, cross-sectional, and controlled study enrolled with male AGA or BPH patients above 18 years old. AGA and BPH patients recruited met the diagnostic criteria according to the guidelines (22, 23). They were diagnosed by experienced dermatologists and urologists, respectively. We investigated 742 AGA patients and 657 BPH patients who visited the department of dermatology and urology in Xiangya Hospital from April 1 to August 15, 2021, by telephone and web-based questionnaires. A total of 457 AGA patients and 397 BPH patients were willing to cooperate with our survey (**Figure 1**).

### Questionnaires

The contents of the questionnaire mainly included (1): the socio-demographic characteristics of the respondents (2); the health status of the subjects themselves (3); the treatment conditions, especially anti-androgen therapies (4); the date and type of the SARS-CoV-2 vaccines inoculated if any (5), the occurrence and severity of adverse reactions if any.

Baseline information such as age, height, weight, education, and marital status was obtained. Regional adverse reactions (such as injection site pain, redness and induration) as well as systemic adverse reactions (somnolence, fever, dizziness, headache, chills, fatigue, myalgia, nausea, vomiting, abdominal pain, diarrhea, itching at other sites, dyspnea, throat swelling, and tachycardia) were asked in sequence during the survey. For patients with adverse reactions, the duration and severity of adverse reactions and whether the symptoms were treated in the outpatient, emergency department or hospitalization were recorded.

### Statistical analysis

Continuous variables, including age, height and weight, were denoted as mean ± standard deviation (SD) and compared with t-test. Pearson’s chi-squared test or Fisher’s exact test was used to contrast the difference of categorical variables, including education, marital status, use of finasteride, co-morbidites, and adverse reactions. Odds ratio (OR) was calculated by regression analysis, and OR_adj_ was obtained after adjusting for confounding factors such as height and weight. SPSS 23 was used for all data analysis. Values of *p* < 0.05 were considered statistically significant.

## Result

### Characteristics of participants

Overall, 457 AGA participants had completed the survey with a mean age of 27.03 ± 6.54 years, and 424 patients (92.8%) had received at least the first dose of vaccine at the time of the survey. There were significant differences in the marital status, use of anti-androgen therapies, and co-morbidities between vaccinated and unvaccinated populations. (**Table 1**).

**Table 1:** Characteristics of AGA and BPH patients vaccinated and unvaccinated for SARS-CoV-2.

	AGA				BPH			
	Total (N=457)	Unvaccinated (N=33)	Vaccinated (N=424)	*p^1^*	Total(N=397)	Unvaccinated (N=155)	Vaccinated (N=242)	*p^2^*	*p*
Age, years old (mean ± SD) [min, max]	27.03 ± 6.54 [18, 56]	28.52 ± 6.38 [20, 42]	26.92 ± 6.55 [18, 56]	0.177	64.13 ± 9.11 [31; 91]	67.47 ± 9.31 [46, 91]	61.99 ± 8.31 [31, 85]	<0.001**	<0.001**
Height, cm (mean ± SD)	172.9 ± 5.39	172.15 ± 4.40	172.86 ± 5.44	0.138	167.26 ± 5.06	167.33 ± 5.20	167.21 ± 4.99	0.838	<0.001**
Weight, kg (mean ± SD)	69.66 ± 10.51	67.44 ± 12.21	70.14 ± 12.23	0.039*	64.49 ± 8.93	63.49 ± 9.56	65.09 ± 8.49	0.122	<0.001**
Education, %				0.463				0.554	<0.001**
Primary/middle school	0	0	0		50.70	51.40	50.30		
high school	3.70	6.10	3.50		27.90	30.30	26.30		
College or above	96.30	93.90	96.50		21.40	18.30	23.40		
Marital Status, %				<0.001**				0.089	<0.001**
Unmarried	71.10	72.90	48.50		0.30	0.70	0		
Married	28.90	27.10	51.50		98.40	96.60	99.60		
Divorced	0	0	0		0.50	0.70	0.40		
Widowed	0	0	0		0.80	2.00	0		
Use of finasteride, %				<0.001**				0.578	<0.001**
Yes	48.80	24.20	53.30		81.40	20.00	17.80		
No	51.20	75.80	46.70		18.60	80.00	82.20		
Therapies other than finasteride				<0.001**				0.851	<0.001**
Yes	57.30	81.80	55.40		93.20	92.90	93.40		
No	42.70	18.20	44.60		6.80	7.10	6.60		
Has co-morbidities				<0.001**				0.195	<0.001**
Yes	33.50	57.60	31.60		54.70	58.70	52.10		
No	66.50	42.40	68.40		45.30	41.30	47.90		

AGA, androgenetic alopecia; BPH, benign prostatic hyperplasia; SD, Standard Deviation; p^1^, p value between the vaccinated and unvaccinated AGA groups; p^2^, p value between the vaccinated and unvaccinated BPH groups; p, p value between AGA and BPH groups; *, p<0.05 indicates significant difference; **, p<0.001 indicates significant difference.

A total of 397 validated questionnaires were received from BPH patients with an average age of 64.13 ± 9.11 years, and 61.0% of them were vaccinated. Vaccinated BPH patients (61.99 ± 8.31) were younger than unvaccinated populations (67.47 ± 9.31). (**Table 1**) AGA tended to present a higher vaccination rate than in BPH patients. (*p* < 0.001).

### Associated factors for unvaccination among AGA and BPH patients

After adjusting for height and weight (**Table 2**), it was found that use of anti-androgen therapy and having co-morbidities significantly increase the risk of unvaccination among AGA patients. Use of anti-androgen therapy, specifically finasteride, affected the willingness of vaccination negatively by more than three times (OR: 3.729; 95% CI: 1.564, 8.891, *p* < 0.001). Furthermore, AGA patients with comorbidities were more reluctant to get inoculated (OR: 2.875; 95% CI: 1.359, 6.984, *p* < 0.001). As for BPH, age was the only significantly associated factor that might decrease vaccine inoculation rate, despite a small scale of negative influence (OR: 1.073; 95% CI:1.041-1.107, *p* < 0.001). In addition, having co-morbidities affected the intention of vaccination negatively with a difference that nearly reached statistical significance (OR: 1.579; 95% CI:0.988-2.524, *p* = 0.056).

**Table 2:** Associated factors for unvaccination among AGA and BPH patients.

	AGA				BPH				
	Unadjusted model		Adjusted model^#^		Unadjusted model		Adjusted model^#^		
	OR (95%CI)	*p*	OR (95%CI)	*p*	OR (95%CI)	*p*	OR (95%CI)	*p*	
Age	1.034 (0.985, 1.085)	0.182	1.044 (0.991, 1.101)	0.106	1.075 (1.048, 1.103)	<0.001**	1.073 (1.041, 1.107)	<0.001**
Education								
Primary/middle school					Ref.			
High school	Ref.				1.126 (0.642, 1.974)	0.678	1.227 (0.676, 2.227)	0.502
College or above	0.570 (0.125, 2.605)	0.468	0.425 (0.089, 2.025)	0.283	0.768 (0.408, 1.447)	0.414	0.963 (0.489, 1.873)	0.897
Use of finasteride	3.585 (1.581,8.130)	<0.001**	3.729 (1.564, 8.891)	<0.001**	1.157 (0.692, 1.933)	0.578	1.134 (0.642, 2.004)	0.664
Co-morbidities	2.927 (1.425, 6.014)	<0.001**	2.875 (1.359, 6.984)	<0.001**	1.309 (0.871, 1.967)	0.195	1.579 (0.988, 2.524)	0.056

AGA, androgenetic alopecia; BPH, benign prostatic hyperplasia; OR, odds ratio; CI, confidence interval.

^#^, adjusted for height and weight.

**, p<0.001 indicates significant difference.Ref. indicates the reference group used for regression analysis.

### Adverse reactions after vaccination by the type of vaccines and treatment

The total incidence of adverse reactions in AGA patients was 31.1% (132/424). Injection site pain was most frequently seen, followed by fatigue and myalgia for AGA patients as shown in **Table 3**. In particular, the incidence of fever, headache, and injection site pain varies among different types of vaccines among AGA patients. Around 9.5% (23/242) BPH patients presented adverse reactions. Similarly, injection site pain was the most frequently observed side effect as shown in **Table 4**.

**Table 3:** Adverse reactions of vaccination by the vaccine types and the treatment among AGA patients (N=424).

Adverse reaction (N, %)	Total	Type of vaccination				Treatment		
		Adenovirus vector vaccine (N=7)	Inactivated vaccine (N=354)	Recombinant subunit vaccine(N=63)	*p*	No use of finasteride(N=262)	Use of finasteride(N=162)	*p*		
Fever	13	3, 42.9%	9, 2.5%	1, 1.6%	<0.001**	6, 2.3%	7, 4.3%	0.239
Dizzy	14	1, 14.3%	13, 3.7%	0	0.084	11, 4.2%	3, 1.9%	0.199
Headache	5	1, 14.3%	4, 1.1%	0	<0.001**	4, 1.5%	1, 0.6%	0.399
Chill	2	0	2, 0.6%	0	0.820	2, 0.8%	0	0.265
Fatigue	47	1, 14.3%	36, 10.2%	10, 15.9%	0.399	33, 12.6%	14, 8.6%	0.208
Myalgia	16	0	12, 3.4%	4, 6.3%	0.456	7, 2.7%	9, 5.6%	0.130
Diarrhea	1	0	1, 0.3%	0	0.906	1, 0.4%	0	0.431
Dyspnea	1	0	1, 0.3%	0	0.906	0	1, 0.6%	0.203
Skin Leision	2	0	1, 0.3%	1, 1.6%	0.373	2, 0.8%	0	0.265
Injection-site Redness	2	0	2, 0.6%	0	0.820	2, 0.8%	0	0.265
Injection-site Induration	4	0	4, 1.1%	0	0.671	3, 1.1%	1, 0.6%	0.585
Injection-site Pain	79	4, 57.1%	64, 18.1%	11, 17.5%	0.030*	42, 16.0%	37, 22.8%	0.080

AGA, androgenetic alopecia; *, p<0.05 indicates significant difference; **, p<0.001 indicates significant difference.

**Table 4:** Adverse reactions of vaccination by the vaccine types and the treatment among BPH patients (N=242).

Adverse reaction (N, %)	Total	Type of vaccination				Treatment		
		Adenovirus vector vaccine (N=12)	Inactivated vaccine(N=200)	Recombinant subunit vaccine(N=30)	*p*	No use of finasteride(N=195)	Use of finasteride(N=47)	*p*		
Dizzy	3	0	2, 1.0%	1, 3.3%	0.517	2, 1.0%	1, 2.1%	0.446
Fatigue	6	0	6, 3.0%	0	0.524	5, 2.6%	1, 2.1%	0.985
Myalgia	2	0	2, 1.0%	0	0.809	2, 1.0%	0	0.521
Injection-site Redness	1	0	1, 0.5%	0	0.900	1, 0.5%	0	0.651
Injection-site Induration	1	0	1, 0.5%	0	0.900	1, 0.5%	0	0.651
Injection-site Pain	14	0	12, 6.0%	2, 6.7%	0.671	13, 6.7%	1, 2.1%	0.314

BPH, benign prostatic hyperplasia.

In populations with anti-androgen therapy among all androgen sensitive phenotypes, fever and headache altered due to vaccine types as shown in **Table 5**. Interestingly, injection site pain presented a lower frequency in those without use of finasteride (*p* = 0.019) (**Table 5**). And use of finasteride increased injection site pain by 1.708 times (95% CI: 1.088 - 2.683, *p* = 0.020).

**Table 5:** Adverse reactions of vaccination by the vaccine types and the treatment among patients with androgen sensitive phenotypes (N=666).

Adverse reaction (N, %)	Total	Type of vaccination				Treatment		
		Adenovirus vector vaccine (N=19)	Inactivated vaccine (N=554)	Recombinant subunit vaccine(N=93)	*p*	No use of finasteride(N=463)	Use of finasteride(N=203)	*p*		
Fever	13	3, 15.8%	9, 1.6%	1, 1.1%	<0.001**	6, 1.3%	7, 3.4%	0.065
Dizzy	17	1, 5.3%	15, 2.7%	1, 1.1%	0.489	13, 2.8%	4, 1.9%	0.528
Headache	5	1, 5.3%	4, 0.7%	0	0.052	4, 0.9%	1, 0.5%	0.609
Chill	2	0	2, 0.4%	0	0.833	2, 0.4%	0	0.348
Fatigue	53	1, 5.3%	42, 7.6%	10, 10.8%	0.525	38, 8.2%	15, 7.4%	0.719
Myalgia	18	0	14, 2.5%	4, 4.3%	0.558	9, 1.9%	9, 4.4%	0.068
Diarrhea	1	0	1, 0.2%	0	0.913	1, 0.2%	0	0.508
Dyspnea	1	0	1, 0.2%	0	0.913	0	1, 0.5%	0.131
Skin Leision	2	0	1, 0.2%	1, 1.1%	0.347	2, 0.4%	0	0.348
Injection-site Redness	3	0	3, 0.5%	0	0.760	3, 0.6%	0	0.250
Injection-site Induration	5	0	5, 0.9%	0	0.632	4, 0.8%	1, 0.5%	0.609
Injection-site Pain	93	4, 21.1%	76, 13.7%	13, 14.0%	0.663	55, 11.9%	38, 18.7%	0.019*

*, p<0.05 indicates significant difference; **, p<0.001 indicates significant difference.

## Discussion

Here, we conducted a cross-sectional, retrospective, single-center controlled study to investigate the potential associated factors for SARS-CoV-2 vaccination and its adverse reactions among AGA and BPH patients. Anti-androgen therapy and coexistence of comorbidities might reduce vaccination rate for AGA patients, while age was discovered as the most important factor that hold back BPH patients from SARS-CoV-2 vaccines. Adverse reactions varied depending on the vaccine types and the usage of anti-androgen therapy.

Health status has been proved to be a major factor affecting vaccination, which is controversial among diverse territories. Several studies in Saudi Arabia presented that vaccination intentions are significantly lower in chronically ill (52%) populations than in the general populations (67%) (24, 25). On the contrary, studies in Europe and the United States revealed that patients with chronic diseases have a relatively high willingness to receive vaccines (26, 27). This phenomenon may attribute to local publicity and education of COVID-19 vaccines. A recent Chinese study (28) discovered that the more the public knew about COVID-19 vaccines, the higher the willingness to be vaccinated. In addition, more and more adverse reactions were reported up to now, such as allergic reactions, which increased public concerns about the safety of the vaccines (29). Patients with systemic diseases might be more cautious to take new vaccines since whether or not it aggravates the primary diseases has not been elucidated. In our study, having comorbidities significantly affected vaccination willingness negatively among AGA patients, which might ascribe to the concerns for side effects. On account of the high risk for infection, popularizing the close relevance between SARS-CoV-2 infection and androgen sensitive phenotypes, especially AGA and BPH, and the protective effects of vaccines, is urgently needed in helping COVID-19 vaccine generalization.

Aging was proposed as a factor contributing to prevent vaccines among BPH patients according to our study. This is identical to the previous survey conducted in Saudi Arabia that the elderly was less willing to be vaccinated (43.85% vs 67%) (25, 30). A possible reason might be the age restriction for newly established SARS-CoV-2 vaccine programs at the initial stage. In addition, the elder population tended to be less awareness of the COVID-19 pandemic and vaccines since they had fewer information sources, which results in a low vaccination rate. The elderly, which takes up an increasing proportion of the population, is more susceptible to infections due to their poor health status and weakened immune conditions. Their health should be paid more attention especially when during the pandemic.

Based on the androgen sensitive mechanism of SARS-CoV-2 infection, amounts of studies have proposed anti-androgen therapy as a promising treatment method. Proxalutamide, an androgen receptor antagonist, reduces hospitalization rates in outpatients with COVID-19 (31). Further, early use of dutasteride (a 5-alpha-reductase inhibitor) was found to relieve COVID-19 symptoms (12). Interestingly, receiving anti-androgen therapy was revealed as an affecting factor holding back AGA patients from SARS-CoV-2 vaccination in our study. It might be owing to the concern of the unknown interactions between SARS-CoV-2 vaccines and anti-androgen therapy. A similar phenomenon was found in cancer patients. Receiving multiple treatments reduced their willingness to vaccinate, in consideration of the drug-vaccine interactions (32).

Though the relationship between anti-androgen therapy and SARS-CoV-2 vaccines has not been revealed, previous researches have discovered a close correlation between androgen deprivation and immune responses induced by other vaccines. Humoral immunity and cell-mediated immunity are classic adaptive immune responses induced by either SARS-CoV-2 infection or vaccination (33–36). The validity of the immune responses determines the symptoms severity of COVID-19 patients and lays the foundation for the efficacy of the vaccine. T cell immune response is a crucial determinant in the control of SARS-CoV-2, which primes in the early stage and contributes to clinical protection (37) CD4^+^ helper T cells help in the elicitation of robust neutralizing antibodies, activation of cytotoxic T cells and formation of memory cells, which are crucial in the regulation of antiviral immunity (38–40). CD8^+^ cytotoxic T cells killed infected cells directly and secreted antiviral cytokines, such as IFN-γ and TNF-α (40, 41). Interestingly, androgen has been revealed as a negative regulator for T cell mediated immune response (42–46). A study presented a phenomenon that women almost universally respond better than men to vaccinations that induce antibodies (42). Androgen deprivation boosted CD8^+^ T cell immune response in male mice (43, 44). Also, ablation of androgen in prostate cancer patients increased T-cell infiltration into tumor tissue (45). Previous research revealed that androgen inhibits murine and human CD4 T cells differentiated into T-helper 1 cells by blocking IL-2 signaling, providing solid evidence for targeting androgen in rescuing immune responses in confronting virus infection (46). Except for T cells, androgens and androgen receptors were found suppressing B cells and dendritic cells through regulating B cell homeostasis (47). Taken together, it was easy to come up with the idea that anti-androgen therapy might serve as a vaccine booster for SARS-CoV-2 adaptive immunity (48). Still, SARS-CoV-2 specific IgG expression levels under anti-androgen therapies warrant more data to reveal the specific influence of anti-androgen therapy on SARS-CoV-2 vaccines. Other than the enhanced influence on adaptive immune response after vaccination, anti-androgen therapy could also decrease the susceptibility for COVID-19 among androgen sensitive phenotypes since the androgen receptor shares the same locus with SARS-COV-2.

Adverse effects of vaccinations have not been fully elucidated. A possible explanation for adverse effects might be undue immune responses (49). Antiandrogen therapy enhances the adaptive immune responses of both T cells and B cells among AGA and BPH patients, which might mediate excessive immune responses and lead to discomforts, such as fever, according to our results. Yet, anti-androgen therapy increased incidence of injection site pain by 1.7 times. This may be correlated with the interaction between anti-androgen drugs and the vaccine medium. SARS-CoV-2 specific IgG levels should be detected among those with anti-androgen therapy to better clarify the association in future research.

There were some limitations in this study. Firstly, there was some recall bias during the survey. Secondly, our population was relatively small and restricted to a single center of patients, which resulted in a selection bias.

In conclusion, the importance of SARS-CoV-2 vaccines should be strengthened and popularized among AGA and BPH despite the comorbidities or use of anti-androgen therapy.

## Data availability statement

The original contributions presented in the study are included in the article/supplementary material. Further inquiries can be directed to the corresponding author.

## Ethics statement

The studies involving human participants were reviewed and approved by Ethics Committee of Xiangya Hospital, Central South University. The patients/participants provided their authorized informed consent to participate in this study.

## Author contributions

Investigation, ZF, SD, ZY, RB, JC, and TL; Resources, FL and HX; Supervision, WS and HX; Writing – original draft, ZF; Writing – review & editing, JL and YT. All authors contributed to the article and approved the submitted version.

## Funding

This work was supported by the National Natural Science Foundation of China (No.82073467).

## Acknowledgments

The assistance of outpatients in Xiangya Hospital to participate in data collection is gratefully acknowledged.

## Conflict of interest

The authors declare that the research was conducted in the absence of any commercial or financial relationships that could be construed as a potential conflict of interest.

## Publisher’s note

All claims expressed in this article are solely those of the authors and do not necessarily represent those of their affiliated organizations, or those of the publisher, the editors and the reviewers. Any product that may be evaluated in this article, or claim that may be made by its manufacturer, is not guaranteed or endorsed by the publisher.

## Abbreviations

COVID-19, Coronavirus disease 2019; SARS-CoV-2, syndrome coronavirus 2; AGA, androgenetic alopecia; BPH, benign prostatic hyperplasia; ACE2, Angiotensin-converting enzyme 2; TMPRSS2, transmembrane protease serine 2; SD, standard deviation; OR, Odds ratio; Cl, confidence interval; DHT, Dihydrotestosterone.
